# Dose escalation results from a first-in-human, phase 1 study of glucocorticoid-induced TNF receptor–related protein agonist AMG 228 in patients with advanced solid tumors

**DOI:** 10.1186/s40425-018-0407-x

**Published:** 2018-09-25

**Authors:** Ben Tran, Richard D. Carvajal, Aurelien Marabelle, Sandip Pravin Patel, Patricia M. LoRusso, Erik Rasmussen, Gloria Juan, Vijay V. Upreti, Courtney Beers, Gataree Ngarmchamnanrith, Patrick Schöffski

**Affiliations:** 1 Department of Medical Oncology, Peter MacCallum Cancer Centre, 305 Grattan Street, Melbourne, VIC 3000 Australia; 2 Department of Medicine, Herbert Irving Comprehensive Cancer Center, Columbia University Medical Center, New York City, New York USA; 3 Département d’Innovation Thérapeutique et d’Essais Précoces, Gustave Roussy, Université Paris-Saclay, Villejuif, France; 4 INSERM U1015, Gustave Roussy, Villejuif, France; 5 Division of Hematology and Medical Oncology, Moores Cancer Center, University of California San Diego, La Jolla, California USA; 6 Department of Medical Oncology, Yale Cancer Center, New Haven, CT USA; 7 Amgen Inc, Thousand Oaks, California USA; 8 Amgen Inc, South San Francisco, California USA; 9 Department of General Medical Oncology, University Hospitals Leuven, Leuven Cancer Institute, Leuven, Belgium

**Keywords:** Glucocorticoid-induced TNFR-related protein, Antibodies, monoclonal, Clinical trial, phase 1, Dose, maximum tolerated, Agonistic antibody

## Abstract

**Background:**

This open-label, first-in-human, phase 1 study evaluated the safety, pharmacokinetics, pharmacodynamics, and maximum tolerated dose (MTD) of AMG 228, an agonistic human IgG1 monoclonal antibody targeting glucocorticoid-induced tumor necrosis factor receptor−related protein (GITR), in patients with refractory advanced solid tumors.

**Methods:**

AMG 228 was administered intravenously every 3 weeks (Q3W). Dose escalation was in two stages: single-patient cohorts (3, 9, 30, and 90 mg), followed by “rolling six” design (*n* = 2–6; 180, 360, 600, 900, and 1200 mg). Primary endpoints included incidence of dose-limiting toxicities (DLTs), AEs, and pharmacokinetics. Additional endpoints were objective response and pharmacodynamic response.

**Results:**

Thirty patients received AMG 228, which was well tolerated up to the maximum planned dose (1200 mg). No DLTs occurred; the MTD was not reached. The most common treatment-related AEs were fatigue (13%), infusion-related reaction (7%), pyrexia (7%), decreased appetite (7%), and hypophosphatemia (7%). Two patients had binding anti−AMG 228 antibodies (one at baseline); no neutralizing antibodies were detected. AMG 228 exhibited target-mediated drug disposition, and serum exposure was approximately dose proportional at 180–1200 mg and greater than dose proportional at 3–1200 mg. Doses > 360 mg Q3W achieved serum trough coverage for 95% in vitro GITR occupancy. Despite GITR coverage in peripheral blood and tumor biopsies, there was no evidence of T-cell activation or anti-tumor activity.

**Conclusions:**

In patients with advanced solid tumors, AMG 228 Q3W was tolerable up to the highest tested dose (1200 mg), exhibited favorable pharmacokinetics, and provided target coverage indicating a pharmacokinetic profile appropriate for longer intervals. However, there was no evidence of T-cell activation or anti-tumor activity with AMG 228 monotherapy.

**Trial registration:**

ClinicalTrials.gov, NCT02437916.

**Electronic supplementary material:**

The online version of this article (10.1186/s40425-018-0407-x) contains supplementary material, which is available to authorized users.

## Background

Glucocorticoid-induced tumor necrosis factor receptor–related protein (GITR) is a TNF receptor superfamily costimulatory molecule expressed primarily by regulatory T cells (Treg), effector T cells, and natural killer cells that inhibits the suppressive activity of Tregs [1–4]. Agonistic antibodies or GITR ligand binding to GITR in concert with T cell receptor (TCR) stimulation causes activation of the MAPK/ERK pathway and NFkB, resulting in augmentation of T cell proliferation and proinflammatory cytokine production and enhanced anti-tumor effector function [5, 6], as well as resistance of CD4^+^ and CD8^+^ T cells to Treg suppression [4]. In tumor models, signaling through GITR has been shown to inhibit Treg proliferation, induce Treg depletion, and cause tumor regression [7–11]. Consequently, GITR has become an attractive therapeutic target, with several GITR agonists in clinical development for the treatment of solid tumors [11].

AMG 228 is an agonistic human IgG1 monoclonal antibody that binds to human GITR. The objectives of the dose escalation of this open-label, first-in-human, phase 1 study were to evaluate the safety, pharmacokinetics, pharmacodynamics, and maximum tolerated dose (MTD) of AMG 228 in patients with advanced solid tumors.

## Methods

### Patients

Patients aged ≥18 years with treatment-refractory, advanced solid tumors (non–small-cell lung cancer, squamous cell carcinoma of the head and neck, melanoma, colorectal cancer, or urothelial cell carcinoma of the bladder), Eastern Cooperative Oncology Group performance status ≤2, life expectancy > 3 months, and adequate hematologic, cardiac, renal, and hepatic function were eligible for the study.

Exclusion criteria included history of second malignancy; current/prior autoimmune diseases or syndromes requiring steroids or immunosuppressive therapy (except vitiligo or resolved childhood asthma/atopy); diverticulitis, peptic ulcer disease, colitis, inflammatory bowel disease, or other gastrointestinal disease within 2 years of study; severe immune-related adverse reactions from checkpoint inhibitors; arterial thrombosis within 6 months of study; antitumor therapy or immune modulators within 28 days of study; systemic radiation therapy (radioactive substance administered systemically) or focal radiotherapy within 28 or 14 days, respectively, of study drug administration. Institutional review board and/or ethics committee approval was obtained for all procedures. Patients provided informed consent.

### Study design and treatment

This open-label, first-in-human phase 1 study (NCT02437916) was conducted at six institutions. The study was designed to investigate the safety and tolerability, immunogenicity, pharmacokinetics, MTD, antitumor activity, and pharmacodynamic response of AMG 228 as monotherapy and was planned with two parts: a two-stage dose escalation (part 1) and a dose expansion (part 2). In the first stage of the dose escalation, single-patient cohorts enrolled sequentially to receive intravenous AMG 228 every 3 weeks (Q3W) at prespecified doses of 3, 9, 30, or 90 mg. After receiving AMG 228, each patient entered a 21-day treatment-free period for the assessment of dose-limiting toxicity (DLT), defined as any grade 3 or 4 treatment-related hematologic or nonhematologic toxicity per Common Terminology Criteria for Adverse Events (CTCAE), version 4.0. After a grade ≥ 2 adverse event (AE) considered related to AMG 228, a DLT, or efficacy in stage 1, using a “rolling six” design [12], multiple-patient cohorts (up to 6 patients each) enrolled in the second stage to receive intravenous AMG 228 Q3W at the prespecified doses of 180, 360, 600, 900, or 1200 mg. After the first 3 patients in each cohort were enrolled, there was a 48-h waiting period before the next patient enrolled. Escalation continued until identification of a preliminary MTD up to the highest planned dose level. The MTD was defined as the maximum dose at which up to 33% of patients experienced a DLT. Dose escalation completed when the highest planned dose was assessed, a Bayesian model [13] predicted the dose at which 6 patients were already enrolled, 50 DLT-evaluable patients were enrolled, or a MTD was identified. The planned dose expansion (part 2) did not enroll due to lack of evidence of T cell activation and observed anti-tumor activity following treatment.

### Safety and immunogenicity

AEs (graded per CTCAE, version 4.0) were recorded for all patients. Blood samples for the assessment of anti–AMG 228 antibodies were collected predose throughout treatment cycles.

### Pharmacokinetics

Blood samples for the assessment of AMG 228 pharmacokinetics were collected predose, at the end of infusion, and postdose over the 3-week dosing interval during treatment cycles. Serum AMG 228 levels were measured using a validated ELISA. The pharmacokinetic and exposure parameters of AMG 228 estimated using non-compartmental methods (WinNonlin, version 6.4) were maximum observed serum concentration (C_max_), area under the concentration versus time curve in a dosing interval τ (AUC_τ_; *τ* = 3 wk), and terminal elimination half-life (t_1/2_) calculated as ln(2)/λz, where λz is the first-order terminal rate constant estimated via linear regression of the terminal log-linear decay.

### Pharmacodynamics

Blood samples for the assessment of biochemical coverage (GITR expression by Treg) and immune modulation (depletion of Treg and increase of cytotoxic T lymphocyte [CTL] numbers and activation) were collected and assessed using a validated flow cytometry assay (IQVIA) by Q^2^ Solutions (Morrisville, NC, USA) at screening; predose and postdose over the 3-week dosing interval during treatment cycles. Two different peripheral blood assay panels were used. Panel one assessed GITR on T, B, and NK cell subsets and HLA-DR and Ki67 on T cell subsets in whole blood: CD14^+^, CD3^+^, CD3^+^CD4^+^CD8^−^, CD3^+^CD4^−^CD8^+^, CD3^−^CD56^+^CD16^+^, CD3^−^CD56^−^CD16^−^, CD3^−^CD56^−^CD16^−^CD20^+^, CD3^+^CD4^+^CD8^−^CD25^+^FoxP3^+^, CD3^+^CD4^+^CD8^−^CD25^+^CD127^lo^. Panel 2 assessed GITR, OX40, PD-1, PD-L1 and Tim3 expression on T cell subsets in whole blood: CD3^+^, CD3^+^CD4^+^CD8^−^, CD4^+^ naïve (CD3^+^CD4^+^CD8^−^CD45RA^+^CD197^+^), CD4^+^ central memory (CD3 + CD4 + CD8^−^CD45RA^−^CD197^+^), CD4^+^ effector memory (CD3^+^CD4^+^CD8^−^CD45RA^−^CD197^−^), CD4^+^ TEMRA (CD3^+^CD4^+^CD8^−^CD45RA^−^CD197^−^), CD3^+^CD4^−^CD8^+^, CD8^+^ naïve (CD3^+^CD4^−^CD8^+^CD45RA^+^CD197^+^), CD8^+^ central memory (CD3^+^CD4^−^CD8^+^CD45RA^−^CD197^+^), CD8^+^ effector memory (CD3^+^CD4^−^CD8^+^CD45RA^−^CD197^−^), and CD4^+^ TEMRA (CD3^+^CD4^+^CD8^−^CD45RA^−^CD197^−^). A fit-for-purpose assay (Myriad RBM, Austin, TX, USA) was used to measure serum soluble GITR (sGITR) and sGITR ligand (sGITRL).

In the rolling six cohorts, available paired tumor biopsies for the assessment of GITR expression by Treg and CTL were collected and assessed by a validated immunohistochemistry assay (Clarient/NeoGenomics Laboratories) at screening; predose in cycle 3 (day 43); and at the end of treatment. The IHC assays were performed using the Dako automated IHC staining platform, and the stained slides were evaluated by a Clarient/NeoGenomics pathologist using a bright field microscope. The specimens were evaluated for GITR staining of neoplastic and infiltrating immune cells. The percentage of cells with positive GITR expression out of all cells in the tumor region was captured. The percentages of CD4^+^ and CD8^+^ T cells are the number of viable immune cells showing partial or complete membrane staining relative to all viable immune cells present in the sample. The percentages of FoxP3+ cells are the number of viable immune cells showing nuclear staining relative to all viable immune cells present in the sample.

### Tumor response

Tumor imaging was done by computed tomography or magnetic resonance imaging per modified immune-related Response Criteria (irRC) [14]. Assessments were performed during initial patient screening (within 28 days of study day 1); 14 days after dosing in cycle 4; every 12 weeks (± 1 week) thereafter; and at the end of treatment.

### Statistics

Primary endpoints were the incidence of DLTs and AEs per patient. Additional endpoints included pharmacokinetics, objective tumor response per irRC, and pharmacodynamic response. Data were summarized descriptively.

## Results

### Patients

Thirty patients with advanced solid tumors were enrolled between April 21, 2015 and August 5, 2016. Patients had relapsed or refractory colorectal cancer (43%), head and neck cancer (33%), urothelial transitional cell carcinoma (13%), non–small-cell lung cancer (7%), or melanoma (3%; Table 1). Most patients (77%) had previously received three or more lines of therapy.

**Table 1:** Demographics and baseline characteristics

Characteristics	All Patients (*N* = 30)
Median age (range), years	63.0 (45.0–83.0)
Sex, n (%)	
Male	19 (63)
Female	11 (37)
Race, n (%)	
White	27 (90)
Black	2 (7)
Unknown	1 (3)
Primary tumor type, n (%)	
Colorectal cancer	13 (43)
Head and neck cancer	10 (33)
Urothelial transitional cell carcinoma	4 (13)
Non–small-cell lung cancer	2 (7)
Melanoma	1 (3)
ECOG performance status, n (%)	
0	9 (30)
1	19 (63)
2	2 (7)
Prior lines of therapy, n (%)	
1	4 (13)
2	3 (10)
≥ 3	23 (77)

*ECOG* Eastern Cooperative Oncology Group

All 30 patients received at least one dose of AMG 228 in the dose escalation phase: 3 mg (*n* = 1), 9 mg (*n* = 1), 30 mg (*n* = 1), 90 mg (*n* = 1), 180 mg (*n* = 6), 360 mg (*n* = 4), 600 mg (*n* = 6), 900 mg (*n* = 4), and 1200 mg (*n* = 6). Reasons for discontinuing treatment were disease progression (*n* = 26), death (*n* = 2), AEs (*n* = 1), and consent withdrawal (*n* = 1).

### Safety and tolerability

No DLTs occurred during the 21-day DLT assessment period, so the MTD was not reached. Thirty (100%) patients experienced treatment-emergent AEs (Table 2), the majority (60%) of which were grade 1 or 2. The most common (occurring in ≥20% of patients) treatment-emergent AEs were fatigue (33%), anemia (27%), nausea (23%), and hypophosphataemia (23%), vomiting (20%), pyrexia (20%), and hypertension (20%). Eighteen (60%) patients had AEs that were considered by the investigators to be related to treatment with AMG 228. The most common (occurring in ≥5% of patients) treatment-related AEs were fatigue (13%), infusion-related reaction (7%), pyrexia (7%), decreased appetite (7%), and hypophosphataemia (7%). Most of the treatment-related AEs were of grade 1 (14 patients [47%]) or grade 2 (six patients [20%]) in severity.

**Table 2:** Incidence of adverse events per patient

	All Patients (*N* = 30)
Patients with any treatment-emergent AE, n (%)	30 (100)
Patients with any treatment-emergent serious AE, n (%)	12 (40)
Patients with a grade 3 treatment-related AE, n (%)	0
Patients with a grade 4 treatment-related AE, n (%)	0
Patients with a grade 5 treatment-related AE, n (%)	1 (3)
Incidence of treatment-related AEs, n (%)	18 (60)
Fatigue, all grades	4 (13)
Grade 1	3 (10)
Grade 2	1 (3)
Infusion-related reaction, all grades	2 (7)
Grade 1	2 (7)
Pyrexia, all grades	2 (7)
Grade 1	2 (7)
Decreased appetite, all grades	2 (7)
Grade 1	2 (7)
Hypophosphataemia, all grades	2 (7)
Grade 2	2 (7)

*AE* adverse event

*AEs occurring in in ≥5% of patients are shown

Overall, twelve (40%) patients had serious AEs. Two (7%) patients had serious AEs that were considered treatment-related. The first patient (1200-mg cohort) with colorectal cancer had serious, grade 2 treatment-related proteinuria on study day 22 that began to resolve 2 days after AMG 228 was withheld. On study day 40 (1 week before progressive disease was confirmed), AMG 228 was permanently discontinued. A second patient (1200-mg cohort) with microsatellite stable colorectal cancer died 30 days after the last dose of AMG 228 because of a serious AE of pulmonary disease labeled as pneumonitis considered possibly related to study AMG 228. Imaging confirmed radiographic disease progression but the patient consented to continue treatment with AMG 228 per the protocol. The cause of death was hypoxia due to pneumonitis; however, lung biopsy was not performed to confirm or rule out the diagnosis. The patient was unresponsive to treatment with steroids and a single dose of infliximab. The investigator reported infection and lymphangitic disease progression of underlying malignant disease as potential contributors. No other patients had AEs resulting in treatment discontinuation.

Three patients had fatal AEs. In addition to the patient with fatal treatment-related pneumonitis, one patient had fatal acute hypoxemic respiratory failure not related to AMG 228, and another patient died from progressive disease.

Postbaseline binding anti−AMG 228 antibodies were detected in two patients, one of whom had positive results at baseline. The antibodies did not appear to affect exposure to AMG 228. No patients had detectable neutralizing anti−AMG 228 antibodies.

### Pharmacokinetics

**Fig. 1:**
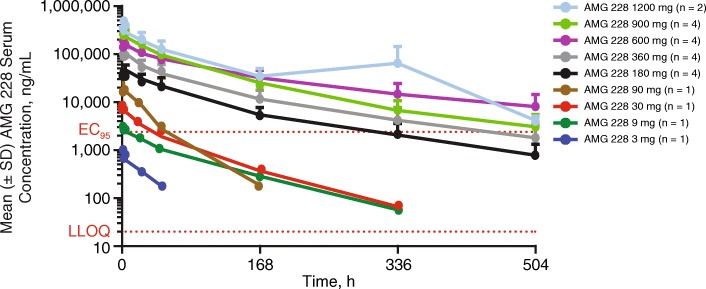
Mean (± SD) pharmacokinetic profile of AMG 228 following intravenous administration of AMG 228 every 3 weeks. Blood samples were collected predose, at the end of infusion, and postdose over the 3-week dosing interval during treatment cycles

AMG 228 pharmacokinetic profiles (Fig. 1) exhibited a pattern consistent with target-mediated drug disposition at lower doses (3–90 mg), as the t_1/2_ was shorter at lower doses of 3–90 mg (0.5–2.9 days) versus the t_1/2_ at higher doses of 180–1200 mg (4.0–5.4 days). Based on a comparison of AUC_τ_ and C_max_ across the entire dose range and higher doses, as well as linear regression analysis of dose-normalized log-transformed AUC_τ_ and C_max_ values, AMG 228 exposure increased in an approximately dose-proportional manner over the higher dose range and in a greater than dose-proportional manner over the entire dose range. No significant serum accumulation of AMG 228 was observed following multiple Q3W doses.

AMG 228 doses > 360 mg resulted in serum trough coverage needed for 95% in vitro receptor occupancy on human peripheral mononuclear cells activated with anti-CD3 antibody for GITR expression (95% effective concentration [EC_95_]).

### Antitumor activity

**Fig. 2:**
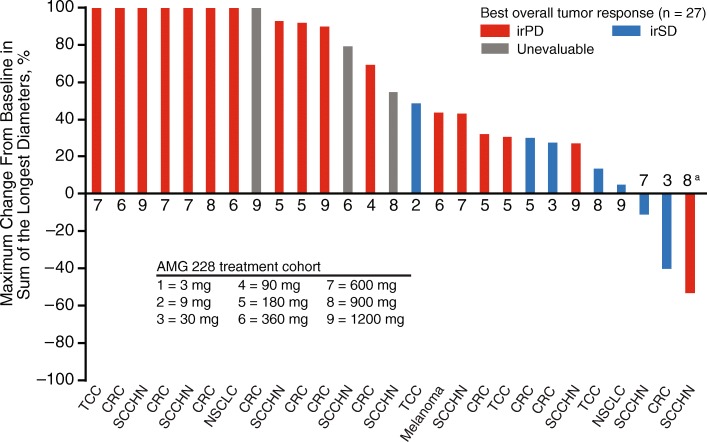
Best change from baseline in the sum of longest diameters of target lesions. CRC, colorectal cancer; HirPD, immune-related progressive disease; irSD, immune-related stable disease. ^a^Patient experienced clinical progression with new brain metastasis (off schedule scan) after the primary lesions showed decreases in size. ^b^Because patient’s only postbaseline scan was an abdominal CT. CRC, colorectal cancer; NSCLC, non–small-cell lung cancer; SCCHN, squamous cell carcinoma of the head and neck; TCC, transitional carcinoma of the bladder

Imaging for the evaluation of tumor response per irRC was available for 27 of 30 patients. Three patients had no evaluable postbaseline scan due to disease progression (*n* = 2) and AE (*n* = 1). No complete or partial responses were observed (Fig. 2). Seven (23%) patients had irRC stable disease, and 17 (57%) had irRC progressive disease. Given the low evidence of clinical activity by AMG 228 monotherapy (consistent with the lack of pharmacodynamic activity; see below *Immunologic response to treatment*), part 2 of the study (dose expansion) was not initiated.

### Immunologic response to treatment

AMG 228 was previously shown to bind with high avidity to human GITR expressed on activated CD4^+^ T cells (mean [SD] half maximal effect concentration [EC_50_], 6.57 [0.57]) and CD8^+^ T cells (mean [SD] EC_50_, 8.75 [1.09]), as measured by flow cytometry (Additional file 1: A). In the presence of anti-CD3 stimulation of TCR, AMG 228 co-stimulated human CD4^+^ T cells in vitro, with EC_50_ values ranging from 0.1433 to 0.9211 ng/mL, depending on the donor (Additional file 1: B). Furthermore, AMG 228 inhibited Treg (CD4^+^CD25^+^FoxP3^high^) suppression of human effector T cells in vitro (Additional file 1: C).

**Fig. 3:**
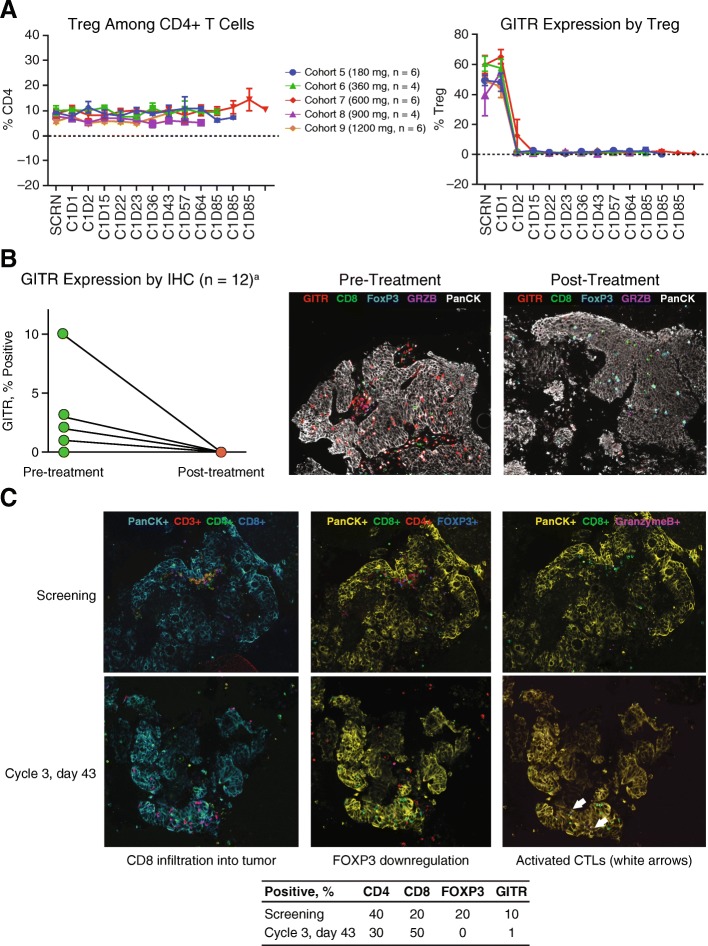
Pharmacodynamic assessment of target coverage and T cell activation. **a** flow cytometry analysis of GITR expression among peripheral blood Tregs pretreatment and posttreatment. **b** immunohistochemistry assessment of GITR expression and T cell activation in tumor biopsies pretreatment and posttreatment. **c** immunohistochemistry evidence of immune modulation following treatment (cycle 3, day 43) versus screening in a colorectal cancer biopsy. ^a^Lines not visible for 7 patients due to lack of GITR^+^ cells at pretreatment

In this first-in-human study, 26 patients had available pre- and post-treatment peripheral blood samples (180 mg, *n* = 6; 360 mg, *n* = 4; 600 mg, *n* = 6; 900 mg, *n* = 4; 1200 mg, *n* = 6) for the assessment of biochemical target coverage and immune modulation. The proportions of Tregs (CD4^+^CD25^+^FoxP3^high^) among CD4^+^ T-cells remained relatively unchanged from screening through treatment cycle 5 (day 85), whereas the proportion of Tregs expressing GITR dropped sharply after 1 day of treatment (Fig. 3a). Serum sGITR and sGITRL were not detected at any timepoint. No notable changes in other peripheral blood lymphocyte populations were observed.

Among 12 patients with available matched paired tumor biopsies (cohorts 5, 6, 7, and 9; *n* = 3 each), GITR expression was detected in pretreatment samples but not in posttreatment samples, and there was generally no evidence of CD8^+^ T-cell infiltration or granzyme B activation posttreatment (Fig. 3b; Additional file 2). However, comparison of pre- and post-treatment tumor biopsies from a patient with advanced colorectal cancer indicated a shift from a suppressive tumor microenvironment at baseline, characterized by Tregs and GITR expression, toward a tumor microenvironment characterized by a lack of Tregs, increased numbers of CD8^+^ T-cell infiltrates, and 10-fold less GITR expression (10% to 1%), indicating tumor target coverage in this patient (Fig. 3c).

## Discussion

Preclinical tumor models have demonstrated the therapeutic potential of targeting GITR with agonistic antibodies [7–11]. In this first-in-human study, AMG 228, an agonistic human IgG1 monoclonal antibody targeting GITR, was generally well tolerated at doses up to the planned maximum dose (1200 mg Q3W) in heavily pretreated patients with refractory solid tumors, and no MTD was identified. AMG 228 − related AEs were generally mild or moderate in severity, with the most frequently occurring (≥5%) events being fatigue, infusion-related reaction, pyrexia, decreased appetite, and hypophosphataemia. No DLTs occurred during the initial 21-day assessment. Similar results have been reported in early clinical studies of other agonistic anti-GITR monoclonal antibodies [15, 16]. In this study, three patients had fatal AEs, two of which were deemed unrelated to study treatment (hypoxemic respiratory failure; progression of malignant melanoma). One patient had fatal pulmonary disease labeled by the investigator as pneumonitis in the setting of progressive underlying cancer but the relationship between this event and AMG 228 could not be ruled out without performing an autopsy.

AMG 228 exposure increased in an approximately dose-proportional manner over the higher dose range and increased in a greater than dose-proportional manner across the entire studied dose range (3 to 1200 mg). Target-mediated drug disposition appeared to affect lower dose cohorts from 3 to 90 mg, possibly contributing to lower exposures. AMG 228 doses above 360 mg resulted in serum trough coverage needed for 95% in vitro receptor occupancy on human PBMCs, suggesting that AMG 228 may have the potential for biologic activity at this dose range. The observed pharmacokinetic profile supported a longer than Q3W dosing interval for AMG 228 in this patient population. The incidence of immunogenicity was low, and binding antibodies did not appear to affect AMG 228 exposure. Overall, AMG 228 exhibited a favorable pharmacokinetic profile in this population.

The decision not to enroll the dose expansion phase of the study was made based on the a lack of evidence of antitumor activity seen during the dose escalation but also considered the inadequate immunologic response, which together, suggested that AMG 228 monotherapy was unlikely to provide meaningful clinical benefit. During the dose escalation, no patients had objective responses, and seven patients (23%) had a best result of immune-related stable disease. Similar findings have been reported among patients with advanced solid tumors treated in a phase 1 first-in-human study of TRX-518, a monoclonal antibody against GITR [15]. However, because objective response is not a primary objective of phase 1 trials, the small sample size and patient characteristics of this heavily pretreated population may have contributed to underestimation of therapeutic effects of AMG 228.

It was hypothesized that treatment with AMG 228 would abrogate the suppressive activity of intratumoral GITR^+^ Treg and enhance activation of GITR^+^ effector T cells, resulting in a shift from a suppressive microenvironment to an effector microenvironment. In mice, the in vivo anti-tumor activity of anti-GITR agonistic antibodies depends on Fc-gamma receptor−dependent, intratumoral depletion of tumor-specific GITR^+^ Tregs [17]. However, despite complete coverage of GITR by AMG 228 in peripheral blood and in tumor biopsies and a decrease in blood GITR^+^ Treg numbers following treatment, little evidence of immune modulation (ie, T cell activation and granzyme B activation) was observed. It is possible, however, that oligomerisation or crosslinking with AMG 228 may have occurred, although the extent is unclear. Also, there were no clinical signs of dose-dependent increase in autoimmune AEs that such Treg depletion should induce in healthy tissues, as observed with anti-CTLA-4 antibodies [18, 19]. Decreased circulating Treg numbers and downregulated FoxP3 expression were observed among patients treated with TRX-518 in a first-in-human study [20]. Studies with immune checkpoint agonists targeting other costimulatory molecules (eg, OX40) have likewise demonstrated shifts toward CTL-rich tumor microenvironments [21, 22].

## Conclusions

In conclusion, AMG 228 showed acceptable safety and favorable pharmacokinetics and serum target coverage as a monotherapy administered at intravenous doses up to 1200 mg Q3W in patients with advanced solid tumors. No evidence of T cell activation or antitumor activity was observed. Although AMG 228 as monotherapy may not provide meaningful clinical benefit, investigation of AMG 228 combined with immunotherapy promoting cytotoxic T cell activation may be worthwhile.

## Additional files

Additional file 1: A, AMG 228 binding to GITR on human CD4^+^ and CD8^+^ T cells. Human PBMC (9 × 10^6^ cells/well) were seeded into anti-CD3–coated 6-well plates for 96 h. Five days later, cells were harvested. Activated human T cells were stained with titrated AMG 228-AF647 or IgG1 isotype control and anti-CD4, anti-CD8, and anti-CD25. Mean fluorescence intensities (MFIs) by AMG 228-AF647 binding were plotted. B, AMG 228 co-stimulation of activated human peripheral CD4^+^ T cells. Primary human CD4^+^ T cells were cultured with sub-optimal concentrations of plate-bound anti-CD3 antibody and a titration of either anti-human GITR clone 9H6 (open circles; parental antibody of AMG 228) or human IgG1 isotype control (filled squares) captured by plate-bound anti-human IgG1 antibody. Cells were pulsed with tritiated thymidine for the last 18 h of a 96-h culture. The data points on the left segment of the x-axis represent average counts per minute (CPM) of T cells plus plate-bound anti-CD3 plus plate-bound anti-human-IgG with no anti-human-GITR/isotype control antibody. Average CPM of quadruplicate wells ± SD (duplicate wells for right panel experiment). The EC_50_ for the first donor (left panel) was 0.1433 ng/mL); the EC_50_ for the second donor (right panel) was 0.9211 ng/mL. C, AMG 228 inhibition of Treg-mediated suppression. Human T_eff_ cells (50,000 cells/well) were co-incubated with Tregs (50,000 cells/well) in the presence of T cell activation beads (Act; Treg Suppression Inspector) and serial titrations of AMG 228 beads or huIgG1 isotype control beads in 200ul/well for 5 days. The highest AMG 228 bead or huIgG1 bead concentration in this graph was 0.8 × 10^6^ beads/well. Cells were pulsed with 1 μCi/well 3H-thymidine during the last 18 h of incubation. Results are expressed as the mean and standard error of the mean (SEM) for duplicate measurements of 3H-thymidine incorporation. (EPS 2209 kb)

Additional file 2: GITR expression by CD4+, CD8+, and FoxP3+ cells. (DOCX 51 kb)

## Abbreviations

AE: Adverse event
AUC_τ_: *τ* = 3 wk, area under the concentration versus time curve in a dosing interval τ
C_max_: Maximum observed serum concentration
CTCAE: Common terminology criteria for adverse events
CTL: Cytotoxic T lymphocyte
DLT: Dose-limiting toxicity
GITR: Glucocorticoid-induced tumor necrosis factor receptor–related protein
irRC: Modified immune-related response criteria
MTD: Maximum tolerated dose
Q3W: Every 3 weeks
sGITR: soluble GITR
sGITRL: soluble GITR ligand
t_1/2_: terminal elimination half-life
Treg: Regulatory T cell
