# Supplementary material for: Dose escalation results from a first-in-human, phase 1 study of glucocorticoid-induced TNF receptor–related protein agonist AMG 228 in patients with advanced solid tumors
Source: J Immunother Cancer. 2018 Sep 25;6:93. doi: 10.1186/s40425-018-0407-x (PMC6156919; doi:10.1186/s40425-018-0407-x)
Supplement: Supplementary file 2 — GITR expression by CD4+, CD8+, and FoxP3+ cells. (DOCX 51 kb) [file 40425_2018_407_MOESM2_ESM.docx]

**Additional File 2. GITR expression by CD4+, CD8+, and FoxP3+ cells**

| **Patient** | **Diagnosis** | **Cohort** | **Visit** | **CD4, % Positive*** | **CD8, % Positive*** | **FoxP3, % Positive*** | **GITR, % Positive*** |
| --- | --- | --- | --- | --- | --- | --- | --- |
| 1 | CRC | 180 mg | Screening C3, D43 | <1 1 | <1 <1 | 3 1 | 0 0 |
| 2 | SCCHN | 180 mg | Screening C3, D43 | Missing <1 | Missing 1 | 1 1 | 1 0 |
| 3 | TCC | 180 mg | Screening C3, D43 | 5 5 | 1 1 | 1 2 | 3 0 |
| 4 | CRC | 360 mg | Screening C3, D43 | 1 1 | 1 1 | 1 2 | 2 0 |
| 5 | SCCHN | 360 mg | Screening C3, D43 | 5 1 | 1 1 | 5 1 | 10 0 |
| 6 | NSCLC | 360 mg | Screening C3, D43 | Missing Missing | 15 10 | 3 3 | 0 0 |
| 7 | SCCHN | 600 mg | Screening C3, D43 | 40  25 | 30 15 | 5 5 | 10 0 |
| 8 | SCCHN | 600 mg | Screening C3, D43 | 3 5 | 5 1 | 5 5 | 3 0 |
| 9 | TCC | 600 mg | Screening C3, D43 | 1 0 | 1 0 | <1 1 | 0 0 |
| 10 | CRC | 1200 mg | Screening C3, D43 | 30 60 | 50  40 | 5 8 | 1 0 |
| 11 | SCCHN | 1200 mg | Screening C3, D43 | 40 60 | 50 40 | 5 15 | 0 0 |
| 12 | CRC | 1200 mg | Screening C3, D43 | 40 30 | 20 50 | 20 0 | 10 1 |

CRC, colorectal cancer; NSCLC, non–small-cell lung cancer; SCCHN, squamous cell carcinoma of the head and neck; TCC, transitional carcinoma of the bladder.

^a^Result of 5% or greater is positive.
